# An Experimentally Informed Evolutionary Model Improves Phylogenetic Fit to Divergent Lactamase Homologs

**DOI:** 10.1093/molbev/msu220

**Published:** 2014-07-24

**Authors:** Jesse D. Bloom

**Affiliations:** Division of Basic Sciences and Computational Biology Program, Fred Hutchinson Cancer Research Center, Seattle, WA

**Keywords:** phylogenetics, deep mutational scanning, lactamase, protein evolution, substitution model

## Abstract

Phylogenetic analyses of molecular data require a quantitative model for how sequences evolve. Traditionally, the details of the site-specific selection that governs sequence evolution are not known a priori, making it challenging to create evolutionary models that adequately capture the heterogeneity of selection at different sites. However, recent advances in high-throughput experiments have made it possible to quantify the effects of all single mutations on gene function. I have previously shown that such high-throughput experiments can be combined with knowledge of underlying mutation rates to create a parameter-free evolutionary model that describes the phylogeny of influenza nucleoprotein far better than commonly used existing models. Here, I extend this work by showing that published experimental data on TEM-1 beta-lactamase (Firnberg E, Labonte JW, Gray JJ, Ostermeier M. 2014. A comprehensive, high-resolution map of a gene’s fitness landscape. *Mol Biol Evol.* 31:1581–1592) can be combined with a few mutation rate parameters to create an evolutionary model that describes beta-lactamase phylogenies much better than most common existing models. This experimentally informed evolutionary model is superior even for homologs that are substantially diverged (about 35% divergence at the protein level) from the TEM-1 parent that was the subject of the experimental study. These results suggest that experimental measurements can inform phylogenetic evolutionary models that are applicable to homologs that span a substantial range of sequence divergence.

## Introduction

Most approaches for the phylogenetic analysis of gene sequences require a quantitative evolutionary model specifying the rate at which each site substitutes from one identity to another. In maximum-likelihood and Bayesian approaches, the evolutionary model is used to calculate the likelihood of the observed sequences given the phylogenetic tree (Felsenstein 1981; Huelsenbeck et al. 2001). In distance-based approaches, the evolutionary model is used to calculate the distances between pairs of sequences (Hasegawa et al. 1985; Saitou and Nei 1987). For all these approaches, inaccurate evolutionary models can lead to errors in inferred phylogenetic properties, including incorrect estimates of evolutionary distances (Halpern and Bruno 1998) and incorrect tree topologies (Felsenstein 1978; Huelsenbeck and Hillis 1993; Lartillot et al. 2007; Rokas and Carroll 2008).

Unfortunately, existing phylogenetic evolutionary models are extreme simplifications of the actual process of mutation and selection that shapes sequence evolution (Thorne et al. 2007). At least two major unrealistic assumptions afflict most of these models. First, in order to make phylogenetic algorithms computationally tractable, it is generally assumed that each site within a gene evolves independently. Second, most widely used evolutionary models compound the first assumption of independence among sites with the second unrealistic assumption that all sites evolve identically—a severely flawed assumption as there is overwhelming evidence that proteins have strong preferences for certain amino acids at specific sites (Halpern and Bruno 1998; Ashenberg et al. 2013). It is the second of these unrealistic assumptions that is addressed by the experimentally informed evolutionary model described here.

A major reason that most phylogenetic evolutionary models assume that sites evolve identically is because it has traditionally been thought that there is insufficient information to do better. In the absence of a priori knowledge about selection on individual sites, the parameters of an evolutionary model must be inferred from the same sequences that are being analyzed phylogenetically. For instance, typical codon-level models infer parameters describing the equilibrium frequencies of different codons, the relative rates of transition and transversion mutations, the relative rates of nonsynonymous and synonymous mutations, and in many cases the shapes of distributions that allow some of these rates to be drawn from several categories (Goldman and Yang 1994; Muse and Gaut 1994; Yang et al. 2000; Kosiol et al. 2007). These parameters can easily be inferred for a single general model that applies to all sites in a gene, but it is much more challenging to infer them separately for each site without overfitting the available sequence data (Posada and Buckley 2004; Rodrigue 2013). Some studies have attempted to bypass this problem by predicting site-specific substitution rates or classifying sites based on knowledge of the protein structure (Thorne et al. 1996; Goldman et al. 1998; Rodrigue et al. 2009; Kleinman et al. 2010; Scherrer et al. 2012)—however, such approaches are limited by the fact that the relationship between protein structure and site-specific selection is complex, and cannot be reliably predicted even by state-of-the-art molecular modeling (Potapov et al. 2009).

A more recent and more promising alternative approach is to infer the site-specific substitution process directly from the sequence data (Lartillot and Philippe 2004; Le et al. 2008; Wang et al. 2008; Rodrigue et al. 2010; Wu et al. 2013). Fully specifying a different substitution process for each site in a maximum-likelihood framework requires inferring a large number of free parameters (there are 19×L parameters for a gene with *L* codons if selection is assumed to act on the amino acid sequence). Therefore, the details of the site-specific substitution process must be inferred without overfitting the finite available data. Two strategies have been successfully employed to do this: Constraining sites to fall in a fixed number of different substitution-model classes (Le et al. 2008; Wang et al. 2008) or using nonparametric Bayesian mixture models that treat the site-specific substitution probabilities as random variables that are integrated over a statistical distribution estimated from the data (Lartillot and Philippe 2004; Rodrigue et al. 2010). Although both of these strategies are somewhat complex, they yield much more nuanced evolutionary models that eliminate some of the problems associated with the unrealistic assumption that sites evolve identically.

Even more recently, a new type of high-throughput experiment has begun to yield data that enables the creation of site-specific evolutionary models without any need to infer site-specific selection from the naturally occurring gene sequences that are the subject of the phylogenetic analysis. This new type of experiment is deep mutational scanning (Fowler et al. 2010; Araya and Fowler 2011), a technique in which a gene is randomly mutagenized and subjected to functional selection in the laboratory, and then deep sequenced to quantify the relative frequencies of mutations before and after selection. In cases where the laboratory selection is sufficiently representative of the gene’s real biological function, these experiments provide information that can be used to approximate the site-specific natural selection on mutations. To date, deep mutational scanning has been used to quantify the impact of most nucleotide or codon mutations to several proteins or protein domains (Fowler et al. 2010; McLaughlin Jr et al. 2012; Traxlmayr et al. 2012; Melamed et al. 2013; Roscoe et al. 2013; Starita et al. 2013; Bloom 2014; Firnberg et al. 2014). For a few of these studies, the experimental coverage of possible mutations is sufficiently comprehensive to define site-specific amino acid preferences for all positions in a gene.

I have previously shown that such experimentally determined site-specific amino acid preferences can be combined with measurements of mutation rates to create a parameter-free evolutionary model that describes the phylogeny of influenza nucleoprotein far better than existing nonsite-specific models that contain numerous free parameters (Bloom 2014). Here, I extend that work by showing that it is also possible to create an experimentally informed evolutionary model for another gene. I do this using deep mutational scanning data published by Firnberg et al. (2014) that quantify the effects of nearly all amino acid mutations on TEM-1 beta-lactamase. In this case, no measurements of mutation rates are available, so I construct an evolutionary model that is informed by the experimentally measured site-specific amino acid preferences and also contains a few free parameters representing the mutation rates. I also augment this model with an additional parameter that reflects the stringency of the site-specific amino acid preferences in natural evolution versus the deep mutational scanning experiment used to measure these preferences. I show that this evolutionary model greatly improves the phylogenetic fit to both TEM and SHV beta-lactamases, the latter of which are substantially diverged (about 35% divergence at the protein level) from the TEM-1 parent that was the subject of the deep mutational scanning by Firnberg et al. (2014). These results generalize previous work on experimentally determined evolutionary models and suggest that site-specific amino acid preferences are sufficiently conserved during evolution to be applicable to gene homologs that span a substantial range of sequence divergence.

## Results

### Evolutionary Model

#### Summary of Evolutionary Model

I have previously described a codon-level phylogenetic evolutionary model for influenza nucleoprotein for which both the site-specific amino acid preferences and the nucleotide mutation rates (assumed to be identical across sites) were determined experimentally (Bloom 2014). This work examines a protein for which the site-specific amino acid preferences have been measured experimentally, but for which the nucleotide mutation rates are unknown. It is therefore necessary to extend the evolutionary model to treat the nucleotide mutation rates as unknown free parameters. Here, I describe this extension.

In the model used here, the rate $P_{r,xy}$ of substitution from codon *x* to some other codon *y* at site *r* is

(1) $$P_{r,xy}=Q_{xy}\times F_{r,xy},$$

where *Q_xy_* denotes the rate of mutation from *x* to *y*, and $F_{r,xy}$ gives the probability that a mutation from *x* to *y* fixes if it occurs. This equation assumes that mutation rates are uniform across sites, and that the selection on mutations is site-specific but site-independent (i.e., the fixation probability at one site is not influenced by mutations at other sites).

#### Fixation Probabilities from Amino Acid Preferences

The fixation probability of a mutation from codon *x* to *y* is assumed to depend only on the encoded amino acids A(x) and A(y), as synonymous mutations are assumed to be selectively neutral. The fixation probabilities $F_{r,xy}$ are defined in terms of the experimentally measured amino acid preferences at site *r*, where $\pi_{r,a}$ denotes the preference for amino acid *a* at site *r*, and the preferences at each site sum to one ($1=\sum_{a}\pi_{r,a}$).

As in previous work (Bloom 2014), I consider two different mathematical relationships between the amino acid preferences and the fixation probabilities. However, novel to this work, I consider a generalization of these relationships that allows the stringency of the amino acid preferences to differ between natural sequence evolution and the deep mutational scanning experiments used to measure $\pi_{r,a}$. Specifically, I take the probability of fixation to depend on ${\left(\pi_{r,a}\right)}^{\beta }$ where *β* is a free parameter (constrained to have values ≥0) that scales the stringency of the amino acid preferences. A value of *β* = 1 implies equally stringent preferences in natural evolution and the deep mutational scanning experiments. A value of β<1 corresponds to less stringent preferences in natural evolution than in the deep mutational scanning experiments, whereas a value of β>1 corresponds to more stringent preferences in natural evolution in the deep mutational scanning experiments. Naively, one might conjecture that *β* will typically have values greater than 1, as laboratory experiments tend to be less stringent than natural evolutionary selection. In the following sections, I will describe testing evolutionary models that constrain *β* = 1 versus models that treat *β* as a free parameter.

With the addition of the stringency parameter *β*, I consider the following relationships between the amino acid preferences and the fixation probabilities. The first relationship derives from considering the amino acid preferences to be directly related to selection coefficients and is a generalization of the ninth equation derived by Halpern and Bruno (1998):
(2)
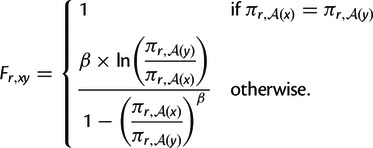
 Note that in equation (2) the mutation terms in the original equation derived by Halpern and Bruno (1998) are all set to be equal as the calculations of the amino acid preferences $\pi_{r,a}$ from the deep mutational scanning experiments already correct for differences in the mutagenesis rates, whereas Halpern and Bruno (1998) were inferring the preferences from natural sequences and so had to account for the mutation rates during natural evolution. Note also that the ninth equation of Halpern and Bruno (1998) contains a typographical error in the denominator which is corrected in equation (2). Halpern and Bruno (1998) derive equation (2) with *β* = 1 by assuming that the sequences are evolving in the weak-mutation limit, and rigorous application of this relationship with *β* = 1 in the context of the current work requires assuming that the effective population size in the deep mutational scanning experiment is equivalent to that for the natural sequences that are being phylogenetically analyzed.

The second relationship is based on considering the amino acid preferences to reflect the fraction of genetic backgrounds that tolerate specific mutations rather than selection coefficients in any one genetic background. Specifically, experiments have shown that mutations that are deleterious in one genetic background can sometimes be neutral (or even advantageous) in a related genetic background (Lunzer et al. 2010; Gong et al. 2013). One reason that mutational effects depend on genetic background is that most proteins are under selection to maintain their overall stability above some critical threshold (Gong et al. 2013). This type of threshold selection gives rise to the evolutionary dynamics described in Bloom et al. (2007), where stabilizing mutations are always tolerated but destabilizing mutations are only tolerated in a fraction of genetic backgrounds. In this case, mutations to higher preference (putatively more stabilizing) amino acids will always be able to fix without deleterious effect, but mutations to lower preference (putatively less stabilizing) amino acids are only sometimes tolerated. One way to represent these dynamics is to use a relationship equivalent to the Metropolis et al. (1953) sampling criterion: (3)
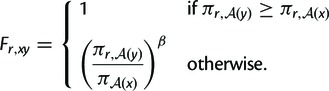



Both of these relationships (eqs. 2 and 3) share the feature that mutations to higher-preference amino acids fix more frequently than mutations to lower-preference amino acids as long as β>0.

#### Mutation Rates

The rate of mutation *Q_xy_* from codon *x* to *y* is defined in terms of the underlying rates of nucleotide mutation. Let *R_m_*_→_*_n_* denote the rate of mutation from nucleotide *m* to *n*. Then (4)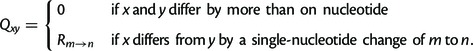 Assuming that the same mutation process operates on both the sequenced and complementary strands of the nucleic acid gives the constraint

(5) $$R_{m\to n}=R_{m_{c}\to n_{c}}$$

where *m*_c_ denotes the complement of nucleotide *m*, as for example a mutation from A to G on one strand induces a mutation from T to C on the other strand. There are therefore six unique nucleotide mutation rates: $R_{\text{A}\to \text{C}}=R_{\text{T}\to \text{G}}$, $R_{\text{A}\to \text{G}}=R_{\text{T}\to \text{C}}$, $R_{\text{A}\to \text{T}}=R_{\text{T}\to \text{A}}$, $R_{\text{C}\to \text{A}}=R_{\text{G}\to \text{T}}$, $R_{\text{C}\to \text{G}}=R_{\text{G}\to \text{C}}$, and $R_{\text{C}\to \text{T}}=R_{\text{G}\to \text{A}}$.

In principle, these six mutation rates could be measured experimentally for the system of interest. In my previous work on influenza nucleoprotein (Bloom 2014), I was able to devise an experimental system for measuring the mutation rates for influenza in cell culture. The mutation rates measured for influenza in this system were roughly symmetric ($R_{m\to n}=R_{n\to m}$), which was sufficient to make the overall evolutionary model in equation (1) reversible. However, in general it is unlikely to be feasible to measure the mutation rates for most systems of interest. Furthermore, it is known that mutation process is AT-biased for many species (Hershberg and Petrov 2010), meaning that in general mutation rates will not be symmetric. Therefore, in general (and also for lactamase specifically) it is necessary to infer the mutation rates from the sequence data without assuming that they are symmetric.

In the absence of any constraints on the six mutation rates given above, the overall evolutionary model defined by equation (1) will not necessarily be reversible. However, it turns out (see Materials and Methods) that placing the following constraint on the mutation rates is sufficient to make the overall evolutionary model reversible:

(6) $$R_{\text{C}\to \text{T}}=\frac{R_{\text{A}\to \text{G}}\times R_{\text{C}\to \text{A}}}{R_{\text{A}\to \text{C}}}.$$

This constraint lacks a biological justification and is assumed purely for the mathematical convenience that it makes the model reversible. Although there is no biological reason to believe that equation (6) actually holds for real evolution, it is possible to give interpretations about what assuming this equation implies about the mutational process. One interpretation is that the probability of mutating from C to G through an intermediate mutation to T is equal to the probability of mutating from C to G through an intermediate mutation to A, as equation (6) implies that $R_{\text{C}\to \text{T}}\times R_{\text{T}\to \text{G}}=R_{\text{C}\to \text{A}}\times R_{\text{A}\to \text{G}}$. Another interpretation is that the AT bias is the same for transitions and transversions, as equation (6) implies that $R_{\text{C}\to \text{T}}/R_{\text{T}\to \text{C}}=R_{\text{C}\to \text{A}}/R_{\text{A}\to \text{C}}$.

In the absence of independent information to calibrate absolute values for the branch lengths or mutation rates, one of the rates is confounded with the branch-length scaling and so can be assigned an arbitrary value greater than 0 without affecting the tree or its likelihood. Here, the choice is made to assign

(7) $$R_{\text{A}\to \text{C}}=1$$

so that all other mutation rates are defined relative to this rate. With these constraints, there are now four independent mutation rates that must be treated as unknown free parameters:

(8) $$\text{unknown mutation rate parameters}=\{\begin{array}{c} R_{\text{A}\to \text{G}}\\ R_{\text{A}\to \text{T}}\\ R_{\text{C}\to \text{A}}\\ R_{\text{C}\to \text{G}} \end{array}$$

In practice, these four mutation rate parameters will be estimated at their maximum-likelihood values given the sequences and tree topology.

#### Equilibrium Frequencies

Calculation of a phylogenetic likelihood requires assigning evolutionary equilibrium frequencies to the possible codons in addition to specifying the transition probabilities given by equation (1). In many conventional phylogenetic models, these equilibrium frequencies are treated as free parameters that are estimated empirically from the sequence data. However, in reality the equilibrium frequencies are the result of mutation and selection, and so can be calculated as the stationary state of the stochastic process defined by the evolutionary model. Specifically, it can be shown (see Materials and Methods) that for the evolutionary model in equation (1), the equilibrium frequency $p_{r,x}$ of codon *x* at site *r* is

(9) $$p_{r,x}=\frac{{\left(\pi_{r,A\left(x\right)}\right)}^{\beta }\times q_{x}}{\sum_{y}{\left(\pi_{r,A\left(y\right)}\right)}^{\beta }\times q_{y}}$$

where *q_x_* is given by (10)
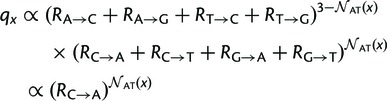
 where $N_{\text{AT}}\left(x\right)$ is the number of A and T nucleotides in codon *x*, the proportionality constant is never needed as *q_x_* values always appear in the form of ratios, and the simplification from the first to second line follows from equations (5)–(7). The equilibrium frequencies $p_{r,x}$ are therefore completely determined by knowledge of the experimentally determined amino acid preferences $\pi_{r,a}$, the mutation rate parameters in equation (8), and the value of the stringency parameter *β*.

### Experimentally Determined Amino Acid Preferences for Beta-Lactamase

The site-specific amino acid preferences for beta-lactamase were determined using data from a previously published deep mutational scanning experiment performed by Firnberg et al. (2014). Specifically, Firnberg et al. (2014) created nearly all possible amino acid mutants of TEM-1 beta-lactamase and then used antibiotic selection to enrich for functional variants at various antibiotic concentrations. Next, they used high-throughput sequencing to examine how the frequencies of mutations changed during this functional selection. They analyzed their data to estimate the impact of individual mutations on TEM-1 function, and had sufficient data to estimate the impact of 96% of the 297 × 19 = 5,453 possible amino acid mutations.

Firnberg et al. (2014) report the impact of mutations in terms of what they refer to as the “fitness” effects. Firnberg et al. (2014) calculate these fitness values from their deep mutational scanning experiment in such a way that a mutation’s fitness effect is approximately proportional to the highest concentration of antibiotic on which bacteria carrying that beta-lactamase variant are able to grow. Therefore, although the fitness values are not calculated in a true population-genetics framework, they certainly reflect effects of specific mutations on the ability of TEM-1 mutants to function.

Here, I use the fitness values provided by Firnberg et al. (2014) to estimate the preferences for each of the 20 amino acids at each site in TEM-1. Specifically, let $w_{r,a}$ be the fitness value for mutation to amino acid *a* at site *r* reported by Firnberg et al. (2014) in supplementary data S2 of their article. I calculate the preference $\pi_{r,a}$ for *a* at site *r* as

(11) $$\pi_{r,a}=\frac{w_{r,a}}{\sum_{a\prime }w_{r,a\prime }}$$

where the sum over a′ ranges over all 20 amino acids, the wild-type amino acid at site *r* is assigned a fitness of $w_{r,a}=1$ in accordance with the normalization scheme used by Firnberg et al. (2014), and the $w_{r,a}$ values for the 4% of mutations for which no value is estimated by Firnberg et al. (2014) are set to the average $w_{r,a}$ of all nonwild-type amino acids at site *r* for which a $w_{r,a}$ value is provided.

**Fig. 1.**
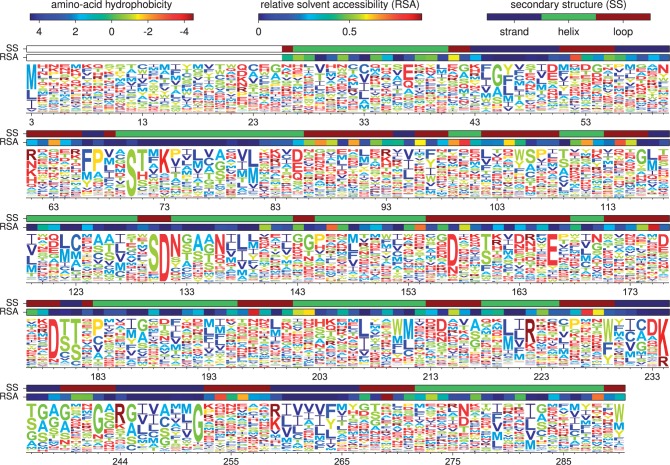
The amino acid preferences for TEM-1 beta-lactamase, calculated from the data of Firnberg et al. (2014). The heights of letters are proportional to the preference for that amino acid at that position in the protein. Residues are numbered using the scheme of Ambler et al. (1991). Letters are colored according to the hydrophobicity of the amino acid. Bars above the letters indicate the secondary structure and relative solvent accessibility as calculated from the crystal structure in PDB entry 1XPB (Fonzé et al. 1995) using DSSP (Kabsch and Sander 1983; Joosten et al. 2011), with maximum solvent accessibilities taken from Tien et al. (2013). The figure was generated using “WebLogo” (Crooks et al. 2004) integrated into the “mapmuts” software package (Bloom 2014). The data and source code used to create this plot are provided through http://jbloom.github.io/phyloExpCM/example_2014Analysis_lactamase.html (last accessed July 28, 2014).

The amino acid preferences calculated in this manner are displayed graphically in figure 1 along with information about residue secondary structure and solvent accessibility (see supplementary file S1, Supplementary Material online, for numerical data). As is extensively discussed by Firnberg et al. (2014) in their original description of the data, these preferences are qualitatively consistent with known information about highly constrained positions in TEM-1, and show the expected qualitative patterns of higher preferences for specific (particularly hydrophobic) amino acids at residues that are buried in the protein’s folded structure. Here, I focus on using these amino acid preferences in a quantitative phylogenetic evolutionary model as described in the next section.

### Experimentally Determined Amino Acid Preferences Improve Phylogenetic Fit

#### TEM and SHV Beta-Lactamase Phylogenetic Trees

To test whether evolutionary models informed by the experimentally determined amino acid preferences are superior to existing alternative models, I compared the fit of various models with beta-lactamase sequence phylogenies. Firnberg et al. (2014) performed their deep mutational scanning on TEM-1 beta-lactamase. There are a large number of TEM beta-lactamases with high sequence identity to TEM-1; the next closest group of lactamases is the SHV beta-lactamases (Bush et al. 1995), which on average have 62% nucleotide and 65% protein identity to TEM beta-lactamases. I assembled a collection of TEM and SHV beta-lactamases from the manually curated Lahey Clinic database (http://www.lahey.org/Studies/, last accessed July 28, 2014). These sequences were aligned to TEM-1, and highly similar sequences (sequences that differed by less than four nucleotides) were removed. The resulting alignment contained 85 beta-lactamase sequences (supplementary file S2, Supplementary Material online), of which 49 were TEM and 36 were SHV.

**Fig. 2.**
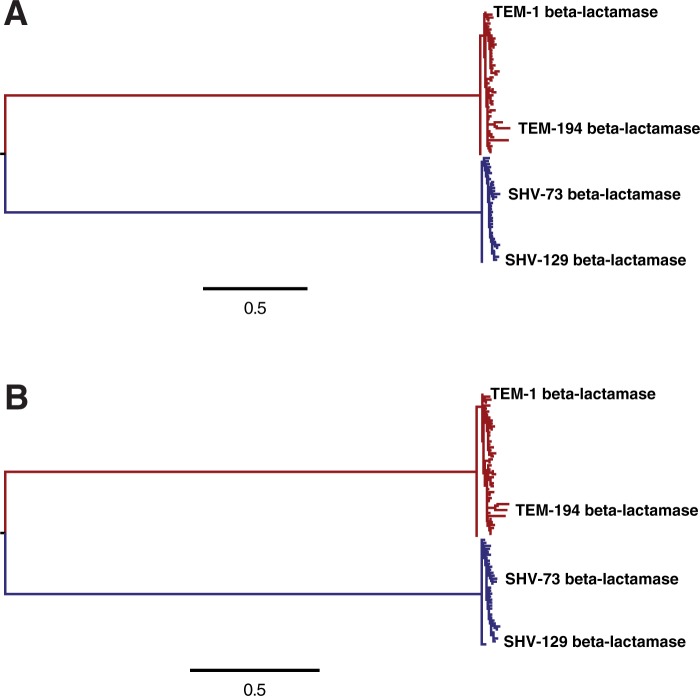
Phylogenetic trees of TEM (red) and SHV (blue) beta-lactamases inferred using “codonPhyML” (Gil et al. 2013) with the codon substitution model of (*A*) Goldman and Yang (1994) or (*B*) Kosiol et al. (2007). The scale bars have units of number of codon substitutions per site. The inferred trees are similar for both models; the distance between the trees computed using the measure of Robinson and Foulds (1981) is 0.14. The TEM and SHV sequences each cluster into closely related clades: The average number of nucleotide and amino acid differences between sequence pairs within these clades is 13 and 7 for the TEM sequences, and 10 and 5 for the SHV sequences. There is extensive divergence between these two clades: The average number of nucleotide and amino acid differences between sequence pairs across the clades is 326 and 100. For both substitution models, a single transition–transversion ratio (*κ*) and four discrete gamma-distributed nonsynonymous–synonymous ratios (*ω*) were estimated by maximum likelihood. The equilibrium codon frequencies were determined empirically using the CF3x4 method (Pond et al. 2010) for the model of Goldman and Yang (1994), or the F method for the model of Kosiol et al. (2007) The data and source code used to create these trees are provided through http://jbloom.github.io/phyloExpCM/example_2014Analysis_lactamase.html (last accessed July 28, 2014).

Maximum-likelihood phylogenetic trees of the TEM and SHV beta-lactamases were constructed using “codonPhyML” (Gil et al. 2013) with the codon substitution model of either Goldman and Yang (1994) or Kosiol et al. (2007). The resulting trees are displayed in figure 2. The two different substitution models give similar tree topologies—the Robinson–Foulds distance (Robinson and Foulds 1981) between the trees inferred with the two different models is calculated by RAxML (Stamatakis 2006) to be 0.14. In both cases, the trees partition into two clades of closely related sequences, corresponding to the TEM and SHV beta-lactamases.

#### Experimentally Informed Models Are Superior for Combined TEM and SHV Phylogeny

To compare the evolutionary models, HYPHY (Pond et al. 2005) was used to optimize the branch lengths and free parameters of the evolutionary models to their maximum-likelihood values on the fixed tree topologies in figure 2. This analysis showed that the evolutionary models informed by the experimentally determined amino acid preferences were clearly superior to commonly used alternative codon-substitution models.

Specifically, tables 1 and 2 show that the experimentally informed evolutionary models fit the combined TEM and SHV phylogeny with higher likelihoods than any of a variety of commonly used alternative models, regardless of which tree topology from figure 2 is used. This superiority is despite the fact that the alternative models (Goldman and Yang 1994; Kosiol et al. 2007) contain many more free parameters. For instance, the most heavily parameterized alternative model contains 60 empirically estimated equilibrium frequency parameters plus an optimized parameter corresponding to the transition–transversion ratio, two optimized parameters corresponding to a gamma distribution of nonsynonymous–synonymous ratios across sites (Yang et al. 2000), and an optimized parameter corresponding to a distribution of substitution rates across sites (Yang 1994). In contrast, the experimentally informed models only contain four or five free parameters (the mutation rates and optionally the stringency parameter *β*)—yet these experimentally informed models have substantially higher likelihoods. When AIC (Posada and Buckley 2004) is used to penalize parameters, the superiority of the experimentally informed models is even more clear.

**Table 1.** Experimentally Informed Evolutionary Models Fit the Combined TEM and SHV Beta-Lactamase Phylogeny in Figure 2*A* Much Better than Models That Do Not Utilize Experimental Data.

Model	ΔAIC	Log Likelihood	Parameters (optimized + empirical)	Optimized Parameters
Experimental, equation (2), free *β*	0.0	–4,020.6	5 (5 + 0)	*R*_A→G_ = 2.3, *R*_A__→__T_ = 0.6, *R*_C__→__A_ = 0.8, *R*_C__→__G_ = 0.7, *β* = 1.6
Experimental, equation (2), *β* = 1	46.4	–4,044.8	4 (4 + 0)	*R*_A__→__G_ = 2.3, *R*_A__→__T_ = 0.6, *R*_C__→__A_ = 0.8, *R*_C__→__G_ = 0.8
Experimental, equation (3), free *β*	77.3	–4,059.3	5 (5 + 0)	*R*_A__→__G_ = 2.2, *R*_A__→__T_ = 0.6, *R*_C__→__A_ = 0.8, *R*_C__→__G_ = 0.7, *β* = 1.2
Experimental, equation (3), *β* = 1	85.7	–4,064.5	4 (4 + 0)	*R*_A__→__G_ = 2.2, *R*_A__→__T_ = 0.6, *R*_C__→__A_ = 0.8, *R*_C__→__G_ = 0.8
GY94, gamma *ω*, gamma rates	392.4	–4,208.8	13 (4 + 9)	*κ* = 2.8, *ω* shape = 0.4, mean *ω* = 0.7, rate shape = 1.2
KOSI07+F, gamma *ω*, gamma rates	410.7	–4,167.0	64 (4 + 60)	*κ* = 0.3, *ω* shape = 0.4, mean *ω* = 4.5, rate shape = 1.4
GY94, gamma *ω*, one rate	460.9	–4,244.1	12 (3 + 9)	*κ* = 2.7, *ω* shape = 0.3, mean *ω* = 1.0
KOSI07+F, gamma *ω*, one rate	467.0	–4,196.1	63 (3 + 60)	*κ* = 0.3, *ω* shape = 0.3, mean *ω* = 5.3
GY94, one *ω*, gamma rates	528.9	–4,278.1	12 (3 + 9)	*κ* = 2.5, *ω* = 0.4, rate shape = 1.2
KOSI07+F, one *ω*, gamma rates	551.3	–4,238.3	63 (3 + 60)	*κ* = 0.4, *ω* = 2.1, rate shape = 1.5
KOSI07+F, one *ω*, one rate	632.9	–4,280.1	62 (2 + 60)	*κ* = 0.4, *ω* = 2.0
GY94, one *ω*, one rate	656.2	–4,342.7	11 (2 + 9)	*κ* = 2.4, *ω* = 0.4
Randomized, equation (3), free *β*	724.5	–4,382.9	5 (5 + 0)	*R*_A__→__G_ = 2.4, *R*_A__→__T_ = 0.6, *R*_C__→__A_ = 0.9, *R*_C__→__G_ = 0.9, *β* = 0.1
Randomized, equation (2), free *β*	735.1	–4,388.2	5 (5 + 0)	*R*_A__→__G_ = 2.4, *R*_A__→__T_ = 0.6, *R*_C__→__A_ = 0.9, *R*_C__→__G_ = 0.9, *β* = 0.0
Avg. frequencies, equation (3), free *β*	820.8	–4,371.0	65 (5 + 60)	*R*_A__→__G_ = 2.4, *R*_A__→__T_ = 0.6, *R*_C__→__A_ = 1.0, *R*_C__→__G_ = 0.9, *β* = 0.5
Avg. frequencies, equation (2), free *β*	841.8	–4,381.5	65 (5 + 60)	*R*_A__→__G_ = 2.4, *R*_A__→__T_ = 0.6, *R*_C__→__A_ = 1.0, *R*_C__→__G_ = 0.9, *β* = 0.3
Avg. frequencies, equation (3), *β* = 1	858.0	–4,390.6	64 (4 + 60)	*R*_A__→__G_ = 2.3, *R*_A__→__T_ = 0.6, *R*_C__→__A_ = 1.1, *R*_C__→__G_ = 1.0
Avg. frequencies, equation (2), *β* = 1	900.7	–4,412.0	64 (4 + 60)	*R*_A__→__G_ = 2.4, *R*_A__→__T_ = 0.6, *R*_C__→__A_ = 1.1, *R*_C__→__G_ = 1.0
Randomized, equation (3)	1,264.9	–4,654.1	4 (4 + 0)	*R*_A__→__G_ = 2.3, *R*_A__→__T_ = 0.6, *R*_C__→__A_ = 0.9, *R*_C__→__G_ = 0.9
Randomized, equation (2)	1,474.5	–4,758.9	4 (4 + 0)	*R*_A__→__G_ = 2.6, *R*_A__→__T_ = 0.6, *R*_C__→__A_ = 0.9, *R*_C__→__G_ = 1.0

Note.—The difference in AIC relative to the best model (smaller ΔAIC indicates better fit), the log likelihood, the number of free parameters, and the values of key parameters are shown in this table. For each model, the branch lengths and model parameters were optimized for the fixed tree topology in figure 2*A*. The “Experimental” models use amino acid preferences derived from the data of Firnberg et al. (2014) plus four mutation rate parameters (eq. 8) and optionally the stringency parameter *β*. For the “Randomized” models, the experimentally measured amino acid preferences are randomized among sites—these models are far worse as the preferences are no longer assigned to the correct positions. For the “Avg. frequencies” models, the amino acid preferences are identical across sites and are set to the average frequency of that amino acid in the entire lactamase sequence alignment—these models are also far worse than the experimentally informed models, as they do not utilize site-specific information. Fitting the stringency parameter to a value of β>1 improves the fit of the experimentally informed models by enhancing the importance of the site-specific amino acid preferences. Fitting the stringency parameter to a value of β<1 improves the fit of the randomized and Avg. frequencies model by effectively equalizing the preferences across amino acids. “GY94” denotes the model of Goldman and Yang (1994) with nine equilibrium frequency parameters calculated using the CF3x4 method (Pond et al. 2010). “KOSI07+F” denotes the model of Kosiol et al. (2007) with 60 equilibrium frequency parameters calculated using the F methods. All variants of GY94 and KOSI07+F have a single transition–transversion ratio (*κ*) estimated by maximum likelihood. Different model variants either have a single nonsynonymous–synonymous ratio (*ω*) or values drawn from four discrete gamma-distributed categories (Yang et al. 2000), and either a single rate or rates drawn from four discrete gamma-distributed categories (Yang 1994). The data and source code used to generate this table are provided through http://jbloom.github.io/phyloExpCM/example_2014Analysis_lactamase.html (last accessed July 28, 2014).

**Table 2.** Experimentally Informed Evolutionary Models Also Provide a Superior Phylogenetic Fit When the Tree Topology Is Estimated Using the Model of Kosiol et al. (2007) Rather than That of Goldman and Yang (1994).

Model	ΔAIC	Log Likelihood	Parameters (optimized + empirical)	Optimized Parameters
Experimental, equation (2), free *β*	0.0	–4,019.9	5 (5 + 0)	*R*_A__→__G_ = 2.2, *R*_A__→__T_ = 0.5, *R*_C__→__A_ = 0.8, *R*_C__→__G_ = 0.8, *β* = 1.6
Experimental, equation (2), *β* = 1	48.3	–4,045.1	4 (4 + 0)	*R*_A__→__G_ = 2.2, *R*_A__→__T_ = 0.5, *R*_C__→__A_ = 0.8, *R*_C__→__G_ = 0.8
Experimental, equation (3), free *β*	76.1	–4,058.0	5 (5 + 0)	*R*_A__→__G_ = 2.1, *R*_A__→__T_ = 0.5, *R*_C__→__A_ = 0.8, *R*_C__→__G_ = 0.8, *β* = 1.3
Experimental, equation (3), *β* = 1	85.6	–4,063.7	4 (4 + 0)	*R*_A__→__G_ = 2.1, *R*_A__→__T_ = 0.5, *R*_C__→__A_ = 0.8, *R*_C__→__G_ = 0.8
GY94, gamma *ω*, gamma rates	398.0	–4,210.9	13 (4 + 9)	*κ* = 2.8, *ω* shape = 0.4, mean *ω* = 0.6, rate shape = 1.3
KOSI07+F, gamma *ω*, gamma rates	402.2	–4,162.0	64 (4 + 60)	*κ* = 0.3, *ω* shape = 0.4, mean *ω* = 4.2, rate shape = 1.4
KOSI07+F, gamma *ω*, one rate	455.1	–4,189.5	63 (3 + 60)	*κ* = 0.3, *ω* shape = 0.4, mean *ω* = 4.3
GY94, gamma *ω*, one rate	464.4	–4,245.1	12 (3 + 9)	*κ* = 2.7, *ω* shape = 0.3, mean *ω* = 0.9
GY94, one *ω*, gamma rates	527.9	–4,276.9	12 (3 + 9)	*κ* = 2.5, *ω* = 0.4, rate shape = 1.2
KOSI07+F, one *ω*, gamma rates	529.8	–4,226.9	63 (3 + 60)	*κ* = 0.4, *ω* = 2.0, rate shape = 1.6
KOSI07+F, one *ω*, one rate	608.3	–4,267.1	62 (2 + 60)	*κ* = 0.4, *ω* = 2.0
GY94, one *ω*, one rate	651.9	–4,339.9	11 (2 + 9)	*κ* = 2.3, *ω* = 0.4
Randomized, equation (3), free *β*	726.3	–4,383.1	5 (5 + 0)	*R*_A__→__G_ = 2.4, *R*_A__→__T_ = 0.5, *R*_C__→__A_ = 0.9, *R*_C__→__G_ = 0.9, *β* = 0.1
Randomized, equation (2), free *β*	737.0	–4,388.4	5 (5 + 0)	*R*_A__→__G_ = 2.4, *R*_A__→__T_ = 0.5, *R*_C__→__A_ = 0.9, *R*_C__→__G_ = 0.9, *β* = 0.0
Avg. frequencies, equation (3), free *β*	823.7	–4,371.8	65 (5 + 60)	*R*_A__→__G_ = 2.4, *R*_A__→__T_ = 0.6, *R*_C__→__A_ = 1.0, *R*_C__→__G_ = 1.0, *β* = 0.5
Avg. frequencies, equation (2), free *β*	844.8	–4,382.3	65 (5 + 60)	*R*_A__→__G_ = 2.4, *R*_A__→__T_ = 0.6, *R*_C__→__A_ = 1.0, *R*_C__→__G_ = 1.0, *β* = 0.3
Avg. frequencies, equation (3), *β* = 1	862.1	–4,392.0	64 (4 + 60)	*R*_A__→__G_ = 2.3, *R*_A__→__T_ = 0.6, *R*_C__→__A_ = 1.1, *R*_C__→__G_ = 1.0
Avg. frequencies, equation (2), *β* = 1	907.1	–4,414.5	64 (4 + 60)	*R*_A__→__G_ = 2.4, *R*_A__→__T_ = 0.6, *R*_C__→__A_ = 1.1, *R*_C__→__G_ = 1.1
Randomized, equation (3)	1,265.1	–4,653.5	4 (4 + 0)	*R*_A__→__G_ = 2.3, *R*_A__→__T_ = 0.5, *R*_C__→__A_ = 0.8, *R*_C__→__G_ = 1.0
Randomized, equation (2)	1,474.1	–4,758.0	4 (4 + 0)	*R*_A__→__G_ = 2.5, *R*_A__→__T_ = 0.5, *R*_C__→__A_ = 0.8, *R*_C__→__G_ = 1.1

Note.—This table differs from table 1 in that the phylogenetic fit is to all TEM and SHV sequences using the tree topology in figure 2*B* rather than that in figure 2*A*.

The experimentally informed models are superior to the nonsite-specific models even when the stringency parameter *β* is fixed to one; however, the phylogenetic fit is substantially enhanced by treating *β* as a free parameter (tables 1 and 2). In all cases, fitting of the stringency parameter yields values of β>1, indicating that natural evolution is more stringent than the experiments in its preferences for specific amino acids. This result is consistent with the notion that selection during real evolution selection is more sensitive than the typical laboratory experiment.

To confirm that the superiority of the experimentally informed models is due to the fact that the deep mutational scanning of Firnberg et al. (2014) captures information about the site-specific amino acid preferences, I tested evolutionary models in which these preferences were randomized among sites or were set to the average frequencies of all amino acids in the lactamase alignments. In the former case, the $\pi_{r,a}$ values are randomly shuffled among sites, where in the latter case $\pi_{r,a}$ for all values of *r* is set to the average frequency of amino acid *a* over the entire lactamase alignment. As can be seen from tables 1 and 2, these nonsite-specific models perform substantially worse than even the simplest versions of the models of Goldman and Yang (1994) and Kosiol et al. (2007). This result shows that the improved performance of the experimentally informed evolutionary models is overwhelmingly due to incorporation of information on the site-specific amino acid preferences rather than better modeling of the mutational process.

#### Experimentally Informed Models Are Superior for Individual TEM and SHV Phylogenies

The foregoing results show that experimentally informed models are superior for describing the combined TEM and SHV beta-lactamase phylogeny. Given that the amino acid preferences were determined by experiments using a TEM-1 parent, it is worth asking whether these preferences accurately describe the evolution of both the TEM and SHV sequences, or whether they more accurately describe the TEM sequences (which are closely related to TEM-1; fig. 2) than the SHV sequences (which only have about 65% protein identity to TEM-1; fig. 2). This question is relevant because the extent to which site-specific amino acid preferences are conserved during protein evolution remains unclear. For instance, although several experimental studies have suggested that such preferences are largely conserved among moderately diverged homologs (Serrano et al. 1993; Ashenberg et al. 2013), a simulation-based study has argued that preferences shift substantially during protein evolution (Pollock et al. 2012; Pollock and Goldstein 2014). If the site-specific amino acid preferences are largely conserved during the divergence of the TEM and SHV sequences, then the experimentally informed models should work well for both these groups—but if the preferences shift rapidly during evolution, then the experimentally informed models should be effective only for the closely related TEM sequences.

To test these competing possibilities, I repeated the analysis in the foregoing section separately for the TEM and SHV clades of the overall phylogenetic tree (the red vs. blue clades in fig. 2). This analysis found that the experimentally informed evolutionary models were clearly superior to all alternative models for the SHV as well as the TEM clade (Tables3,  4, 5, and 6). In fact, the extent of superiority of the experimentally informed model (as quantified by AIC) was greater for the SHV clade than the TEM clade, despite the fact that the former has fewer sequences. These results suggest that the applicability of the experimentally determined amino acid preferences extends to beta-lactamase homologs that are substantially diverged from the TEM-1 parent that was the specific subject of the experiments of Firnberg et al. (2014).

**Table 3.** Experimentally Informed Evolutionary Models Also Provide a Superior Phylogenetic Fit When the Analysis Is Limited Only to TEM Beta-Lactamase Sequences.

Model	ΔAIC	Log Likelihood	Parameters (optimized + empirical)	Optimized Parameters
Experimental, equation (2), free *β*	0.0	–2,374.3	5 (5 + 0)	*R*_A__→__G_ = 1.9, *R*_A__→__T_ = 0.4, *R*_C__→__A_ = 1.1, *R*_C__→__G_ = 0.5, *β* = 1.5
Experimental, equation (2), *β* = 1	23.1	–2,386.8	4 (4 + 0)	*R*_A__→__G_ = 1.9, *R*_A__→__T_ = 0.4, *R*_C__→__A_ = 1.1, *R*_C__→__G_ = 0.5
Experimental, equation (3), free *β*	81.8	–2,415.2	5 (5 + 0)	*R*_A__→__G_ = 1.8, *R*_A__→__T_ = 0.4, *R*_C__→__A_ = 1.1, *R*_C__→__G_ = 0.5, *β* = 1.2
Experimental, equation (3), *β* = 1	83.6	–2,417.1	4 (4 + 0)	*R*_A__→__G_ = 1.8, *R*_A__→__T_ = 0.4, *R*_C__→__A_ = 1.1, *R*_C__→__G_ = 0.5
GY94, gamma *ω*, gamma rates	252.2	–2,492.4	13 (4 + 9)	*κ* = 3.1, *ω* shape = 0.3, mean *ω* = 1.3, rate shape = 0.4
GY94, one *ω*, gamma rates	317.6	–2,526.1	12 (3 + 9)	*κ* = 3.0, *ω* = 1.1, rate shape = 0.3
GY94, gamma *ω*, one rate	318.5	–2,526.5	12 (3 + 9)	*κ* = 3.2, *ω* shape = 0.2, mean *ω* = 1.6
KOSI07+F, gamma *ω*, gamma rates	326.9	–2,478.7	64 (4 + 60)	*κ* = 0.3, *ω* shape = 0.3, mean *ω* = 10.4, rate shape = 0.4
KOSI07+F, gamma *ω*, one rate	394.9	–2,513.8	63 (3 + 60)	*κ* = 0.3, *ω* shape = 0.2, mean *ω* = 13.7
KOSI07+F, one *ω*, gamma rates	412.0	–2,522.3	63 (3 + 60)	*κ* = 0.3, *ω* = 7.9, rate shape = 0.3
Randomized, equation (3), free *β*	465.8	–2,607.2	5 (5 + 0)	*R*_A__→__G_ = 2.1, *R*_A__→__T_ = 0.5, *R*_C__→__A_ = 1.2, *R*_C__→__G_ = 0.5, *β* = 0.0
Randomized, equation (2), free *β*	466.2	–2,607.4	5 (5 + 0)	*R*_A__→__G_ = 2.1, *R*_A__→__T_ = 0.5, *R*_C__→__A_ = 1.2, *R*_C__→__G_ = 0.5, *β* = 0.0
GY94, one *ω*, one rate	483.3	–2,609.9	11 (2 + 9)	*κ* = 2.9, *ω* = 1.1
KOSI07+F, one *ω*, one rate	556.7	–2,595.7	62 (2 + 60)	*κ* = 0.3, *ω* = 7.2
Avg. frequencies, equation (2), free *β*	574.6	–2,601.6	65 (5 + 60)	*R*_A__→__G_ = 2.1, *R*_A__→__T_ = 0.5, *R*_C__→__A_ = 1.2, *R*_C__→__G_ = 0.5, *β* = 0.3
Avg. frequencies, equation (3), free *β*	577.9	–2,603.2	65 (5 + 60)	*R*_A__→__G_ = 2.1, *R*_A__→__T_ = 0.5, *R*_C__→__A_ = 1.2, *R*_C__→__G_ = 0.5, *β* = 0.3
Avg. frequencies, equation (2), *β* = 1	609.1	–2,619.8	64 (4 + 60)	*R*_A__→__G_ = 2.1, *R*_A__→__T_ = 0.5, *R*_C__→__A_ = 1.3, *R*_C__→__G_ = 0.6
Avg. frequencies, equation (3), *β* = 1	622.7	–2,626.7	64 (4 + 60)	*R*_A__→__G_ = 2.0, *R*_A__→__T_ = 0.5, *R*_C__→__A_ = 1.3, *R*_C__→__G_ = 0.6
Randomized, equation (3)	976.6	–2,863.6	4 (4 + 0)	*R*_A__→__G_ = 2.0, *R*_A__→__T_ = 0.5, *R*_C__→__A_ = 1.1, *R*_C__→__G_ = 0.5
Randomized, equation (2)	1,007.8	–2,879.2	4 (4 + 0)	*R*_A__→__G_ = 2.3, *R*_A__→__T_ = 0.5, *R*_C__→__A_ = 1.1, *R*_C__→__G_ = 0.5

Note.—This table differs from table 1 in that the phylogenetic fit is only to the TEM sequences (the portion of the tree shown in red in fig. 2*A*).

**Table 4.** Experimentally Informed Evolutionary Models Also Provide a Superior Phylogenetic Fit When the Analysis Is Limited Only to SHV Beta-Lactamase Sequences.

Model	ΔAIC	Log Likelihood	Parameters (optimized + empirical)	Optimized Parameters
Experimental, equation (2), free *β*	0.0	–1,728.5	5 (5 + 0)	*R*_A__→__G_ = 4.4, *R*_A__→__T_ = 1.9, *R*_C__→__A_ = 0.5, *R*_C__→__G_ = 1.1, *β* = 2.5
Experimental, equation (3), free *β*	34.9	–1,746.0	5 (5 + 0)	*R*_A__→__G_ = 4.2, *R*_A__→__T_ = 1.9, *R*_C__→__A_ = 0.5, *R*_C__→__G_ = 1.1, *β* = 2.2
Experimental, equation (2), *β* = 1	106.2	–1,782.7	4 (4 + 0)	*R*_A__→__G_ = 4.5, *R*_A__→__T_ = 1.9, *R*_C__→__A_ = 0.6, *R*_C__→__G_ = 1.2
Experimental, equation (3), *β* = 1	116.5	–1,787.8	4 (4 + 0)	*R*_A__→__G_ = 4.4, *R*_A__→__T_ = 1.9, *R*_C__→__A_ = 0.6, *R*_C__→__G_ = 1.2
KOSI07+F, gamma *ω*, gamma rates	489.0	–1,914.0	64 (4 + 60)	*κ* = 0.2, *ω* shape = 0.5, mean *ω* = 2.9, rate shape = 0.2
KOSI07+F, one *ω*, gamma rates	499.7	–1,920.4	63 (3 + 60)	*κ* = 0.2, *ω* = 1.8, rate shape = 0.3
GY94, gamma *ω*, gamma rates	505.8	–1,973.4	13 (4 + 9)	*κ* = 3.6, *ω* shape = 0.6, mean *ω* = 0.5, rate shape = 0.2
GY94, one *ω*, gamma rates	514.0	–1,978.5	12 (3 + 9)	*κ* = 3.4, *ω* = 0.3, rate shape = 0.2
KOSI07+F, gamma *ω*, one rate	555.7	–1,948.4	63 (3 + 60)	*κ* = 0.3, *ω* shape = 0.3, mean *ω* = 2.0
KOSI07+F, one *ω*, one rate	573.6	–1,958.3	62 (2 + 60)	*κ* = 0.3, *ω* = 1.8
GY94, gamma *ω*, one rate	581.5	–2,012.3	12 (3 + 9)	*κ* = 3.4, *ω* shape = 0.3, mean *ω* = 0.4
Randomized, equation (3), free *β*	601.7	–2,029.4	5 (5 + 0)	*R*_A__→__G_ = 5.0, *R*_A__→__T_ = 1.8, *R*_C__→__A_ = 0.6, *R*_C__→__G_ = 1.4, *β* = 0.1
GY94, one *ω*, one rate	602.6	–2,023.8	11 (2 + 9)	*κ* = 3.4, *ω* = 0.3
Randomized, equation (2), free *β*	602.7	–2,029.9	5 (5 + 0)	*R*_A__→__G_ = 5.1, *R*_A__→__T_ = 1.8, *R*_C__→__A_ = 0.6, *R*_C__→__G_ = 1.5, *β* = 0.0
Avg. frequencies, equation (3), free *β*	711.5	–2,024.3	65 (5 + 60)	*R*_A__→__G_ = 5.0, *R*_A__→__T_ = 2.0, *R*_C__→__A_ = 0.7, *R*_C__→__G_ = 1.5, *β* = 0.3
Avg. frequencies, equation (2), free *β*	715.7	–2,026.4	65 (5 + 60)	*R*_A__→__G_ = 5.1, *R*_A__→__T_ = 1.9, *R*_C__→__A_ = 0.7, *R*_C__→__G_ = 1.5, *β* = 0.3
Avg. frequencies, equation (3), *β* = 1	749.8	–2,044.5	64 (4 + 60)	*R*_A__→__G_ = 4.9, *R*_A__→__T_ = 2.1, *R*_C__→__A_ = 0.8, *R*_C__→__G_ = 1.6
Avg. frequencies, equation (2), *β* = 1	758.8	–2,048.9	64 (4 + 60)	*R*_A__→__G_ = 5.2, *R*_A__→__T_ = 2.1, *R*_C__→__A_ = 0.8, *R*_C__→__G_ = 1.7
Randomized, equation (3)	1,047.0	–2,253.1	4 (4 + 0)	*R*_A__→__G_ = 4.8, *R*_A__→__T_ = 1.8, *R*_C__→__A_ = 0.6, *R*_C__→__G_ = 1.4
Randomized, equation (2)	1,071.2	–2,265.2	4 (4 + 0)	*R*_A__→__G_ = 5.3, *R*_A__→__T_ = 1.7, *R*_C__→__A_ = 0.6, *R*_C__→__G_ = 1.5

Note.—This table differs from table 1 in that the phylogenetic fit is only to the SHV sequences (the portion of the tree shown in blue in fig. 2*A*).

**Table 5.** Experimentally Informed Evolutionary Models Also Provide a Superior Phylogenetic Fit to the TEM Beta-Lactamases When the Tree Topology Is Estimated Using the Model of Kosiol et al. (2007) Rather than That of Goldman and Yang (1994).

Model	ΔAIC	Log Likelihood	Parameters (optimized + empirical)	Optimized Parameters
Experimental, equation (2), free *β*	0.0	–2,378.5	5 (5 + 0)	*R*_A__→__G_ = 1.9, *R*_A__→__T_ = 0.5, *R*_C__→__A_ = 1.1, *R*_C__→__G_ = 0.4, *β* = 1.5
Experimental, equation (2), *β* = 1	25.4	–2,392.2	4 (4 + 0)	*R*_A__→__G_ = 1.9, *R*_A__→__T_ = 0.5, *R*_C__→__A_ = 1.1, *R*_C__→__G_ = 0.5
Experimental, equation (3), free *β*	80.6	–2,418.8	5 (5 + 0)	*R*_A__→__G_ = 1.8, *R*_A__→__T_ = 0.5, *R*_C__→__A_ = 1.1, *R*_C__→__G_ = 0.4, *β* = 1.2
Experimental, equation (3), *β* = 1	83.7	–2,421.4	4 (4 + 0)	*R*_A__→__G_ = 1.8, *R*_A__→__T_ = 0.5, *R*_C__→__A_ = 1.1, *R*_C__→__G_ = 0.4
GY94, gamma *ω*, gamma rates	257.6	–2,499.3	13 (4 + 9)	*κ* = 3.1, *ω* shape = 0.4, mean *ω* = 1.2, rate shape = 0.4
GY94, one *ω*, gamma rates	317.7	–2,530.4	12 (3 + 9)	*κ* = 3.0, *ω* = 1.0, rate shape = 0.3
GY94, gamma *ω*, one rate	324.1	–2,533.6	12 (3 + 9)	*κ* = 3.1, *ω* shape = 0.2, mean *ω* = 1.5
KOSI07+F, gamma *ω*, gamma rates	325.7	–2,482.4	64 (4 + 60)	*κ* = 0.3, *ω* shape = 0.3, mean *ω* = 9.6, rate shape = 0.4
KOSI07+F, gamma *ω*, one rate	393.5	–2,517.3	63 (3 + 60)	*κ* = 0.3, *ω* shape = 0.2, mean *ω* = 12.1
KOSI07+F, one *ω*, gamma rates	402.4	–2,521.7	63 (3 + 60)	*κ* = 0.3, *ω* = 7.4, rate shape = 0.3
Randomized, equation (3), free *β*	472.0	–2,614.5	5 (5 + 0)	*R*_A__→__G_ = 2.1, *R*_A__→__T_ = 0.5, *R*_C__→__A_ = 1.2, *R*_C__→__G_ = 0.5, *β* = 0.0
Randomized, equation (2), free *β*	472.4	–2,614.7	5 (5 + 0)	*R*_A__→__G_ = 2.1, *R*_A__→__T_ = 0.5, *R*_C__→__A_ = 1.2, *R*_C__→__G_ = 0.5, *β* = 0.0
GY94, one *ω*, one rate	488.0	–2,616.5	11 (2 + 9)	*κ* = 2.9, *ω* = 1.0
KOSI07+F, one *ω*, one rate	550.4	–2,596.7	62 (2 + 60)	*κ* = 0.3, *ω* = 6.7
Avg. frequencies, equation (2), free *β*	581.6	–2,609.3	65 (5 + 60)	*R*_A__→__G_ = 2.1, *R*_A__→__T_ = 0.5, *R*_C__→__A_ = 1.2, *R*_C__→__G_ = 0.5, *β* = 0.3
Avg. frequencies, equation (3), free *β*	584.2	–2,610.6	65 (5 + 60)	*R*_A__→__G_ = 2.1, *R*_A__→__T_ = 0.5, *R*_C__→__A_ = 1.2, *R*_C__→__G_ = 0.5, *β* = 0.3
Avg. frequencies, equation (2), *β* = 1	617.3	–2,628.2	64 (4 + 60)	*R*_A__→__G_ = 2.1, *R*_A__→__T_ = 0.5, *R*_C__→__A_ = 1.3, *R*_C__→__G_ = 0.6
Avg. frequencies, equation (3), *β* = 1	629.2	–2,634.1	64 (4 + 60)	*R*_A__→__G_ = 2.0, *R*_A__→__T_ = 0.5, *R*_C__→__A_ = 1.3, *R*_C__→__G_ = 0.6
Randomized, equation (3)	980.7	–2,869.9	4 (4 + 0)	*R*_A__→__G_ = 2.1, *R*_A__→__T_ = 0.5, *R*_C__→__A_ = 1.1, *R*_C__→__G_ = 0.5
Randomized, equation (2)	1,014.6	–2,886.8	4 (4 + 0)	*R*_A__→__G_ = 2.3, *R*_A__→__T_ = 0.5, *R*_C__→__A_ = 1.1, *R*_C__→__G_ = 0.5

**Fig. 3.**
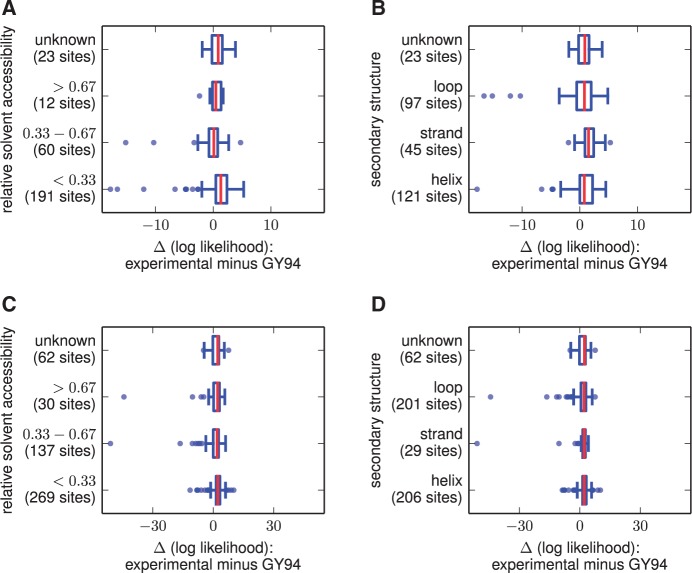
Comparison of likelihoods on a per-site basis between the best experimentally informed site-specific evolutionary model and the best conventional nonsite-specific model. The experimentally informed models are slightly better (positive Δ(log
likelihood)) for most sites, but far worse for a handful of sites. (*A*, *B*) The best experimentally informed lactamase model in table 1 versus the best GY94 variant in table 1. The experimentally informed model has a higher log likelihood for 72% of lactamase sites. (*C*, *D*) The best experimentally informed nucleoprotein model in table 7 versus the best GY94 variant in table 7. The experimentally informed model has a higher log likelihood for 82% of sites. For both genes, the per-site likelihoods were computed after fixing the model parameters and branch lengths to their maximum-likelihood values for the entire gene. Sites are classified in terms of their relative solvent accessibility or secondary structure as computed using DSSP (Kabsch and Sander 1983; Joosten et al. 2011) from PDB structures 1XPB (Fonzé et al. 1995) or 2IQH (Ye et al. 2006), normalizing solvent accessibilities to the values provided by Tien et al. (2013). The per-residue numerical data are in supplementary files S3 and S4, Supplementary Material online. The code and data used to create this figure are provided through http://jbloom.github.io/phyloExpCM/example_2014Analysis_lactamase.html and http://jbloom.github.io/phyloExpCM/example_2014Analysis_Influenza_NP_Human_1918_Descended_withbeta.html (last accessed July 28, 2014).

Note.—This table differs from figure 3 in that the phylogenetic fit is to the TEM sequences using the red portion of tree topology in figure 2*B* rather than the red portion of the tree topology in figure 2*A*.

**Table 6.** Experimentally Informed Evolutionary Models Also Provide a Superior Phylogenetic Fit to the SHV Beta-Lactamases When the Tree Topology Is Estimated Using the Model of Kosiol et al. (2007) Rather than That of Goldman and Yang (1994).

Model	ΔAIC	Log Likelihood	Parameters (optimized + empirical)	Optimized Parameters
Experimental, equation (2), free *β*	0.0	–1,725.4	5 (5 + 0)	*R*_A__→__G_ = 4.4, *R*_A__→__T_ = 1.5, *R*_C__→__A_ = 0.5, *R*_C__→__G_ = 1.3, *β* = 2.4
Experimental, equation (3), free *β*	34.1	–1,742.4	5 (5 + 0)	*R*_A__→__G_ = 4.2, *R*_A__→__T_ = 1.6, *R*_C__→__A_ = 0.5, *R*_C__→__G_ = 1.2, *β* = 2.2
Experimental, equation (2), *β* = 1	104.4	–1,778.5	4 (4 + 0)	*R*_A__→__G_ = 4.4, *R*_A__→__T_ = 1.5, *R*_C__→__A_ = 0.6, *R*_C__→__G_ = 1.3
Experimental, equation (3), *β* = 1	114.6	–1,783.7	4 (4 + 0)	*R*_A__→__G_ = 4.3, *R*_A__→__T_ = 1.6, *R*_C__→__A_ = 0.6, *R*_C__→__G_ = 1.3
KOSI07+F, gamma *ω*, gamma rates	486.6	–1,909.6	64 (4 + 60)	*κ* = 0.2, *ω* shape = 0.8, mean *ω* = 2.1, rate shape = 0.3
KOSI07+F, one *ω*, gamma rates	491.7	–1,913.2	63 (3 + 60)	*κ* = 0.3, *ω* = 1.7, rate shape = 0.3
GY94, gamma *ω*, gamma rates	497.3	–1,966.0	13 (4 + 9)	*κ* = 3.4, *ω* shape = 1.0, mean *ω* = 0.4, rate shape = 0.2
GY94, one *ω*, gamma rates	501.5	–1,969.1	12 (3 + 9)	*κ* = 3.4, *ω* = 0.3, rate shape = 0.2
KOSI07+F, gamma *ω*, one rate	547.6	–1,941.2	63 (3 + 60)	*κ* = 0.3, *ω* shape = 0.3, mean *ω* = 1.9
KOSI07+F, one *ω*, one rate	562.5	–1,949.6	62 (2 + 60)	*κ* = 0.3, *ω* = 1.7
GY94, gamma *ω*, one rate	568.0	–2,002.4	12 (3 + 9)	*κ* = 3.3, *ω* shape = 0.3, mean *ω* = 0.4
GY94, one *ω*, one rate	586.2	–2,012.5	11 (2 + 9)	*κ* = 3.3, *ω* = 0.3
Randomized, equation (3), free *β*	597.7	–2,024.2	5 (5 + 0)	*R*_A__→__G_ = 5.0, *R*_A__→__T_ = 1.5, *R*_C__→__A_ = 0.6, *R*_C__→__G_ = 1.6, *β* = 0.1
Randomized, equation (2), free *β*	598.7	–2,024.7	5 (5 + 0)	*R*_A__→__G_ = 5.0, *R*_A__→__T_ = 1.5, *R*_C__→__A_ = 0.6, *R*_C__→__G_ = 1.6, *β* = 0.0
Avg. frequencies, equation (3), free *β*	706.4	–2,018.6	65 (5 + 60)	*R*_A__→__G_ = 5.0, *R*_A__→__T_ = 1.6, *R*_C__→__A_ = 0.7, *R*_C__→__G_ = 1.7, *β* = 0.3
Avg. frequencies, equation (2), free *β*	710.9	–2,020.8	65 (5 + 60)	*R*_A__→__G_ = 5.0, *R*_A__→__T_ = 1.6, *R*_C__→__A_ = 0.7, *R*_C__→__G_ = 1.7, *β* = 0.3
Avg. frequencies, equation (3), *β* = 1	745.9	–2,039.3	64 (4 + 60)	*R*_A__→__G_ = 4.9, *R*_A__→__T_ = 1.8, *R*_C__→__A_ = 0.8, *R*_C__→__G_ = 1.8
Avg. frequencies, equation (2), *β* = 1	755.3	–2,044.0	64 (4 + 60)	*R*_A__→__G_ = 5.1, *R*_A__→__T_ = 1.7, *R*_C__→__A_ = 0.8, *R*_C__→__G_ = 1.9
Randomized, equation (3)	1,040.9	–2,246.8	4 (4 + 0)	*R*_A__→__G_ = 4.7, *R*_A__→__T_ = 1.5, *R*_C__→__A_ = 0.6, *R*_C__→__G_ = 1.5
Randomized, equation (2)	1,063.9	–2,258.3	4 (4 + 0)	*R*_A__→__G_ = 5.3, *R*_A__→__T_ = 1.4, *R*_C__→__A_ = 0.6, *R*_C__→__G_ = 1.7

Note.—This table differs from table 4 in that the phylogenetic fit is to the SHV sequences using the blue portion of tree topology in figure 2*B* rather than the blue portion of the tree topology in figure 2*A*.

#### The Stringency Parameter β Generally Improves Experimentally Informed Models

The results in the foregoing sections show that use of the stringency parameter *β* improves the phylogenetic fit of the experimentally informed models of lactamase evolution. Previous work (Bloom 2014) has shown that an experimentally informed evolutionary model without a stringency parameter (*β* = 1) improves the phylogenetic fit to influenza nucleoprotein sequences relative to nonsite-specific models. To test whether the fitting of a stringency parameter further improves the experimentally informed evolutionary model of nucleoprotein, I repeated the analysis of Bloom (2014) but also included a model variant in which *β* was fit by maximum likelihood.

Table 7 shows that fitting the stringency parameter substantially improves the phylogenetic fit of the experimentally informed model of nucleoprotein evolution. This table also shows that as with the lactamase models described above, the fitted value of *β* was greater than 1. Overall, this result suggests that inclusion of a stringency parameter generally improves the phylogenetic fit of experimentally informed evolutionary models. Presumably this improvement arises from the fact that deep mutational scanning experiments are generally less stringent than real evolution, and so values of β>1 help scale the experimentally determined site-specific amino acid preferences to a stringency more reflective of those that constrain real evolution.

**Table 7.** Fitting of the Stringency Parameter *β* Also Improves the Phylogenetic Fit of an Experimentally Informed Evolutionary Model for Influenza Nucleoprotein.

Model	ΔAIC	Log Likelihood	Parameters (optimized + empirical)	Optimized Parameters
Experimental, equation (3), free *β*	0.0	–12,144.2	1 (1 + 0)	*β* = 1.7
Experimental, equation (3), *β* = 1	391.8	–12,341.1	0 (0 + 0)	None
GY94, gamma *ω*, gamma rates	1,453.0	–12,858.7	13 (4 + 9)	*κ* = 5.9, *ω* shape = 0.2, mean *ω* = 0.2, rate shape = 2.5
GY94, gamma *ω*, one rate	1,616.0	–12,941.2	12 (3 + 9)	*κ* = 5.6, *ω* shape = 0.2, mean *ω* = 0.2
KOSI07+F, gamma *ω*, gamma rates	1,845.6	–13,004.0	64 (4 + 60)	*κ* = 0.2, *ω* shape = 0.2, mean *ω* = 0.9, rate shape = 1.8
GY94, one *ω*, gamma rates	1,884.1	–13,075.3	12 (3 + 9)	*κ* = 5.9, *ω* = 0.1, rate shape = 2.0
GY94, one *ω*, one rate	2,153.2	–13,210.8	11 (2 + 9)	*κ* = 5.5, *ω* = 0.1
KOSI07+F, gamma *ω*, one rate	2,153.6	–13,159.0	63 (3 + 60)	*κ* = 0.2, *ω* shape = 0.2, mean *ω* = 1.2
KOSI07+F, one *ω*, gamma rates	2,227.3	–13,195.9	63 (3 + 60)	*κ* = 0.2, *ω* = 0.7, rate shape = 1.6
KOSI07+F, one *ω*, one rate	2,650.1	–13,408.3	62 (2 + 60)	*κ* = 0.2, *ω* = 0.7
Avg. frequencies, equation (3), free *β*	3,736.2	–13,952.3	61 (1 + 60)	*β* = 1.2
Avg. frequencies, equation (3), *β* = 1	3,742.2	–13,956.3	60 (0 + 60)	None

Note.—The data in this table were generated by exactly repeating the analysis in the sixth table of Bloom (2014) except including the additional models listed here, which include a model with a stringency parameter. With the exception of the stringency parameter, the experimentally informed evolutionary model used here is derived entirely from experimental measurements, as both the mutation rates and amino acid preferences were measured in Bloom (2014). Only the model using fixation probabilities calculated from equation (3) is reported, as Bloom (2014) shows that this is the best model for influenza nucleoprotein. The data and source code used to generate this table are available through http://jbloom.github.io/phyloExpCM/example_2014Analysis_Influenza_NP_Human_1918_Descended_withbeta.html (last accessed July 28, 2014).

#### Experimentally Informed Models Are Slightly Better for Many Sites

The results described above and in Bloom (2014) demonstrate that experimentally informed evolutionary models improve phylogenetic relative to nonsite-specific models when analyzing the entire gene sequences of lactamase or nucleoprotein. It is interesting to investigate which sites are described more accurately by the experimentally informed models. This can be done by examining the differences in per-site likelihoods between models after fixing the branch lengths and model parameters to their maximum-likelihood values for the entire gene.

Figure 3 compares the per-site likelihoods for the best experimentally informed evolutionary model for lactamase and for nucleoprotein relative to the best nonsite-specific model for each of these genes (using the models from tables 1 and 7). For both lactamase and nucleoprotein, the experimentally informed models lead to small improvements for many sites. Overall, 72% (207 of 286) lactamase sites are described better by the experimentally informed model (supplementary file S3, Supplementary Material online), and 82% (407 of 498) of nucleoprotein sites are described better by the experimentally informed model (4). There appears to be a slight trend for improvements due to the experimentally informed models to be most common for buried sites, but the experimentally informed models lead to small improvements for sites spanning a range of solvent accessibilities and secondary structures.

Interestingly, for both genes there are also a few sites for which the experimentally informed models are far worse than the nonsite-specific models. Presumably, these sites are modeled poorly because the preferences determined by the deep mutational scanning experiments do not accurately capture the real preferences of natural evolution. Such discrepancies could arise from either a failure of the deep mutational scanning experiments to fully reflect natural selection pressures or epistatic interactions that strongly shift the site preferences during natural evolution away from those measured for the parent gene by the experiments. I was unable to observe any obvious features of the specific sites that were described poorly by the experimentally informed models (supplementary files S3 and S4, Supplementary Material online). It therefore remains an open question why certain sites are described very poorly by the experimentally informed models even though the vast majority of sites are described better by these models.

#### Comparison of Different Methods for Computing Fixation Probabilities

In the foregoing analyses, two different mathematical relationships were used to mathematically relate the experimentally determined amino acid preferences to the substitution probabilities in the evolutionary models. One relationship (eq. 2) is based on a true population-genetics derivation by Halpern and Bruno (1998) under the assumption that the preferences are reflective of selection coefficients for amino acids at specific sites (an assumption that would only be strictly true only in the unlikely case that individual sites in a gene contribute independently to fitness). The other relationship (eq. 3) is a more ad hoc one that I suggested in previous work (Bloom 2014) on the grounds that the amino acid preferences might be best envisioned not as selection coefficients, but rather as measurements of the fraction of genetic backgrounds that tolerate a specific mutation, as would be implied by the evolutionary dynamics described in Bloom et al. (2007). Although both relationships share the qualitative feature that mutations to higher-preference amino acids are favored over mutations to lower-preference ones, they differ in their quantitative details. In previous work on influenza nucleoprotein (Bloom 2014), I reported that the relationship in equation (3) outperformed the one in equation (2) derived by Halpern and Bruno (1998).

In contrast, for the beta-lactamase sequences studied here, the relationship of Halpern and Bruno (1998) outperforms the one in equation (3) (tables 1–6). The reason for and relevance of these discordant results remain unclear. There are almost certainly differences in the evolutionary conditions (population size, degree of polymorphism, etc.) for influenza nucleoprotein and beta-lactamase that influence the relationship between selection coefficients and fixation probabilities. In addition, there are substantial differences between the experiments of Firnberg et al. (2014) on beta-lactamase and my previous work on nucleoprotein—in particular, Firnberg et al. (2014) examine the effects of single mutations to the parental gene, whereas the nucleoprotein experiments examined the average effects of individual mutations in variants that often contained multiple mutations. Finally, the experimental measurements are imperfect—for nucleoprotein, the preferences determined by independent biological replicates of the experiments only had a Pearson correlation coefficient of 0.79; Firnberg et al. (2014) do not provide data on the consistency of their measurements across biological replicates, but it seems safe to assume that their experiments are also imperfect. Therefore, further work is probably needed to determine if any meaning can be ascribed to the differences in fit for equation (2) versus equation (3), as well as to identify the optimal mathematical relationship for connecting experimentally measured amino acid preferences to substitution probabilities in evolutionary models. However, both the results presented here and in Bloom (2014) strongly suggest that using any reasonable mathematical relationship to inform evolutionary models with experimentally determined amino acid preferences is sufficient to lead to dramatic improvements in phylogenetic fit.

It is also interesting to speculate on the precise meaning of the stringency parameter *β*. According to population-genetics theory, the strength of selection increases with effective population size. It is therefore tempting to interpret *β* as reflecting differences in the effective population size in the deep mutational scanning experiments relative to natural evolution. Indeed, one could imagine even attempting to use the inferred value of *β* to indirectly quantify effective population size. However, it is important to temper this tantalizing possibility with the reminder that the fixation probabilities over the entire phylogeny can be related to the fitness effects of specific mutations only under the unrealistic assumption that the fitness contributions of mutations at different sites are entirely independent, as these probabilities reflect the effect of a mutation averaged over all genetic backgrounds in the phylogeny. So although the fact that using β > 1 consistently improves phylogenetic does indicate that natural evolution is more stringent in its site-specific amino acid preferences than the experiments, the exact population-genetics interpretation of *β* is unclear.

## Discussion

I have shown that an evolutionary model informed by experimentally determined site-specific amino acid preferences fits beta-lactamase phylogenies better than a variety of existing models that do not utilize site-specific information. When considered in combination with prior work demonstrating that an experimentally determined evolutionary model dramatically improves phylogenetic fit for influenza nucleoprotein (Bloom 2014), these results suggest that experimentally informed models are generally superior to nonsite-specific models for phylogenetic analyses of protein-coding genes. The explanation for this superiority is obvious: Proteins have strong preferences for certain amino acids at specific sites (Halpern and Bruno 1998; Ashenberg et al. 2013) which are ignored by nonsite-specific models. The use of experimentally measured site-specific amino acid preferences improves evolutionary models by informing them about the complex selection that shapes actual sequence evolution. Interestingly, inclusion of a parameter that allows the stringency of site-specific amino acid preferences during natural evolution to exceed those measured by experiments further improves phylogenetic fit, suggesting that deep mutational scanning experiments remain less sensitive than actual natural selection.

I have not compared the experimentally informed evolutionary models with more recent site-specific models that infer aspects of the substitution process from the sequence data itself (Lartillot and Philippe 2004; Le et al. 2008; Wang et al. 2008; Rodrigue et al. 2010; Wu et al. 2013). Such a comparison is desirable as these site-specific models will almost certainly compare more favorably with the experimentally informed models used here. Unfortunately, such a comparison is sufficiently technically challenging to be beyond the scope of this work, as it requires comparing between maximum-likelihood and Bayesian approaches. In any case, the experimentally informed evolutionary models described here should not be viewed purely as competitors for existing site-specific substitution models. Rather, one can imagine future approaches that integrate the results of a deep mutational scanning experiment with additional site-specific details inferred from natural sequence data to create even more nuanced evolutionary models.

An advantage of experimentally informed evolutionary models is that they naturally lend themselves to interpretation in terms of quantities that can be directly related to both specific biochemical measurements and the underlying processes of mutation and selection. This stands in contrast to most existing models, which are phrased in terms of heuristic parameters (such as codon “equilibrium frequencies”) that reflect the combined action of mutation and selection and are not accessible to direct experimental measurement. Experimentally informed evolutionary models therefore have the potential to facilitate connections between the phylogenetic substitution processes and the underlying biochemistry and population genetics of gene evolution (Halpern and Bruno 1998; Thorne et al. 2007).

The major drawback of experimentally informed models is their more limited scope. Most existing codon-based evolutionary models can be applied to any gene (Goldman and Yang 1994; Muse and Gaut 1994; Kosiol et al. 2007)—but experimentally informed models require experimental data for the gene in question. However, this requirement may not be as crippling as it initially appears. The first experimentally determined evolutionary model for influenza nucleoprotein required direct measurement of both the site-specific amino acid preferences and the underlying mutation rates (Bloom 2014). However, the model presented here only requires measurement of the amino acid preferences, as the mutation rates are treated as free parameters. Rapid advances in the experimental technique of deep mutational scanning are making such data available for an increasing number of proteins (Fowler et al. 2010; McLaughlin Jr et al. 2012; Traxlmayr et al. 2012; Melamed et al. 2013; Roscoe et al. 2013; Starita et al. 2013; Bloom 2014; Firnberg et al. 2014).

In this respect, it is encouraging that the site-specific amino acid preferences determined experimentally for TEM-1 improve phylogenetic fit to substantially diverged (35% protein sequence divergence) SHV beta-lactamases as well as highly similar TEM beta-lactamases. As discussed in the Introduction, there are two major limitations to most existing evolutionary models: They treat sites identically, and they treat sites independently. Experimentally informed evolutionary models of the type described here have the potential to completely remedy the first limitation as deep mutational scanning defines site-specific selection with increasing precision. However, such models still treat sites independently—and this limitation will never be completely overcome by experiments, as the unforgiving math of combinatorics means that no experiment can examine all arbitrary combinations of mutations (e.g., TEM-1 has only 5,453 single amino acid mutants, but it has 14,815,801 double mutants, 26,742,520,805 triple mutants, and over 10^13^ quadruple mutants). The utility of experimentally informed evolutionary models therefore depends on the extent to which site-specific amino acid preferences measured for one protein can be extrapolated to other homologs—in other words, are sites sufficiently independent that the preferences at a given position remain similar after mutations at other positions? This question remains a topic of active debate, with experimental studies suggesting that site-specific preferences are largely conserved among closely and moderately related homologs (Serrano et al. 1993; Ashenberg et al. 2013), but some computational studies emphasizing substantial shifts in preferences during evolution (Pollock et al. 2012; Pollock and Goldstein 2014). The fact that the TEM-1 experimental data inform a model that accurately describes the substantially diverged SHV homologs suggests reasonable conservation of site-specific amino acid preferences among beta-lactamase homologs.

This apparent conservation of site-specific amino acid preferences suggests that the phylogenetic utility of experimentally informed evolutionary models may extend well beyond the immediate proteins that were experimentally characterized. This type of experimental generalization would have precedent: Only a tiny fraction of proteins have been crystallized, but because structure is largely conserved during protein evolution, it is frequently possible to use a structure determined for one protein to draw insights about a range of related homologs (Lesk and Chothia 1980; Sander and Schneider 1991). It seems plausible that the conservation of site-specific amino acid preferences could similarly enable deep mutational scanning to provide the experimental data to inform evolutionary models of sufficient scope to improve the accuracy and interpretability of phylogenetic analyses for a substantial number of proteins of interest.

## Materials and Methods

### Availability of Computer Code and Data

The phylogenetic analyses were performed using the software package phyloExpCM (**phylo**genetic analyses with **exp**erimental **c**odon **m**odels, https://github.com/jbloom/phyloExpCM, last accessed July 28, 2014), which primarily serves as an interface to run HYPHY (Pond et al. 2005). Input data, computer code, and a description sufficient to enable replication of all analyses reported in this article are available through http://jbloom.github.io/phyloExpCM/example_2014Analysis_lactamase.html and http://jbloom.github.io/phyloExpCM/example_2014Analysis_Influenza_NP_Human_1918_Descended_withbeta.html (last accessed July 28, 2014).

### Equilibrium Frequencies and Reversibility

Here I show that the evolutionary model defined by equation (1) is reversible (satisfies detailed balance), and has $p_{r,x}$ defined by equation (9) as its equilibrium frequency.

First, note that the fixation probabilities $F_{r,xy}$ defined by both equations (2) and (3) satisfy reversibility with respect to the stringency-adjusted amino acid preferences ${\left(\pi_{r,A\left(y\right)}\right)}^{\beta }$—namely that

(12) $${\left(\pi_{r,A\left(x\right)}\right)}^{\beta }\times F_{r,xy}={\left(\pi_{r,A\left(y\right)}\right)}^{\beta }\times F_{r,yx},$$

as can be verified by direct substitution. This relationship means that if all codon interchanges were equally likely (all *Q_xy_* values are equal), then the equilibrium frequency $p_{r,x}$ of codon *x* would simply be proportional to the stringency-adjusted preference ${\left(\pi_{r,A\left(x\right)}\right)}^{\beta }$ for the encoded amino acid.

However, in practice all codon interchanges are not equally likely, so the actual equilibrium frequencies $p_{r,x}$ will also depend on the mutation rate parameters *R_m_*_→_*_n_* listed in equation (8). This dependence is given by the *q_x_* terms in equation (9). These *q_x_* terms can be thought of as the expected equilibrium frequencies of the codons in a hypothetical situation in which there is no selection and all 64 codons are equally fit (all $F_{r,xy}$ values are equal). In other words, the *q_x_* terms define the stationary state of the reversible stochastic process defined by the mutation rates *Q_xy_*. In order to show *q_x_* given equation (10) defines this stationary state, it is necessary to show that

(13) $$q_{x}\times Q_{xy}=q_{y}\times Q_{yx}.$$

There are up to 12 possible types of single-nucleotide mutations that can be made to a codon *x* to create a different codon *y* (three possible mutations to each of the four possible nucleotides); however, only four of these types of mutations require independent verification of equation (13). Specifically, the possible types of mutations that require independent verification of equation (13) are when *x* differs from *y* by a mutation of A→T, of C→G, of A→C, or of A→G. The other eight types of mutations do not have to be verified because they are equivalent to one of these first four cases: C→A is equivalent to A→C by symmetry (i.e., interchange of the labels of codons *x* and *y*), G→A is equivalent to A→G by symmetry, T→A is equivalent to A→T by symmetry, G→C is equivalent to C→G by symmetry, T→G is equivalent to A→C because of equation (5), T→C is equivalent to A→G because of equation (5), G→T is equivalent to C→A by equation (5) and then to A→C by symmetry, and C→T is equivalent to G→A by equation (5) and then A→G by symmetry. So below I verify equation (13) for the four independent types of mutations, full verifying equation (13).

The first case is where *x* differs from *y* by a mutation of A→T, such that $Q_{xy}=R_{\text{A}\to \text{T}}$ and $Q_{yx}=R_{\text{T}\to \text{A}}$. In this case, we have

(14) $$\begin{array}{c} \;q_{x}\times Q_{xy}={\left(R_{\text{C}\to \text{A}}\right)}^{N_{\text{AT}}\left(x\right)}\times R_{\text{A}\to \text{T}}\\ ={\left(R_{\text{C}\to \text{A}}\right)}^{N_{\text{AT}}\left(y\right)}\times R_{\text{T}\to \text{A}}\\ \;=q_{y}\times Q_{yx}, \end{array}$$

where the first line substitutes the definitions of equations (10) and (4), the second line follows from equation (5) and the fact that $N_{\text{AT}}\left(x\right)=N_{\text{AT}}\left(y\right)$ if *x* and *y* differ only by an A→T mutation, and the final line again substitutes the definitions of equations (10) and (4).

The second case is where *x* differs from *y* by a mutation of C→G, such that $Q_{xy}=R_{\text{C}\to \text{G}}$ and $Q_{yx}=R_{\text{G}\to \text{C}}$. In this case, we have

(15) $$\begin{array}{c} \;q_{x}\times Q_{xy}={\left(R_{\text{C}\to \text{A}}\right)}^{N_{\text{AT}}\left(x\right)}\times R_{\text{C}\to \text{G}}\\ ={\left(R_{\text{C}\to \text{A}}\right)}^{N_{\text{AT}}\left(y\right)}\times R_{\text{G}\to \text{C}}\\ \;=q_{y}\times Q_{yx}, \end{array}$$

where the justifications for the three lines are identical for those used for equation (14).

The third case is where *x* differs from *y* by a mutation of A→C, such that $Q_{xy}=R_{\text{A}\to \text{C}}$ and $Q_{yx}=R_{\text{C}\to \text{A}}$. In this case, we have

(16) $$\begin{array}{c} \;q_{x}\times Q_{xy}={\left(R_{\text{C}\to \text{A}}\right)}^{N_{\text{AT}}\left(x\right)}\times R_{\text{A}\to \text{C}}\\ \;={\left(R_{\text{C}\to \text{A}}\right)}^{N_{\text{AT}}\left(x\right)}\\ ={\left(R_{\text{C}\to \text{A}}\right)}^{N_{\text{AT}}\left(x\right)-1}\times R_{\text{C}\to \text{A}}\\ \;={\left(R_{\text{C}\to \text{A}}\right)}^{N_{\text{AT}}\left(y\right)}\times R_{\text{C}\to \text{A}}\\ \;=q_{y}\times Q_{yx}, \end{array}$$

where the first line substitutes the definitions of equations (10) and (4), the second line uses equation (7), the third line is simple algebra, the fourth line follows from the fact that $N_{\text{AT}}\left(x\right)-1=N_{\text{AT}}\left(y\right)$ if *x* can be converted to *y* by an A→C mutation, and the final line again substitutes the definitions of equations (10) and (4).

The fourth and final case is where *x* differs from *y* by a mutation of A→G, such that $Q_{xy}=R_{\text{A}\to \text{G}}$ and $Q_{yx}=R_{\text{G}\to \text{A}}$. In this case, we have

(17) $$\begin{array}{c} \;q_{x}\times Q_{xy}={\left(R_{\text{C}\to \text{A}}\right)}^{N_{\text{AT}}\left(x\right)}\times R_{\text{A}\to \text{G}}\\ \;={\left(R_{\text{C}\to \text{A}}\right)}^{N_{\text{AT}}\left(x\right)}\times \frac{R_{\text{C}\to \text{T}}}{R_{\text{C}\to \text{A}}}\\ \;={\left(R_{\text{C}\to \text{A}}\right)}^{N_{\text{AT}}\left(x\right)-1}\times R_{\text{C}\to \text{T}}\\ \;={\left(R_{\text{C}\to \text{A}}\right)}^{N_{\text{AT}}\left(y\right)}\times R_{\text{C}\to \text{T}}\\ \;={\left(R_{\text{C}\to \text{A}}\right)}^{N_{\text{AT}}\left(y\right)}\times R_{\text{G}\to \text{A}}\\ \;=q_{y}\times Q_{yx}, \end{array}$$

where the first line substitutes the definitions of equations (10) and (4), the second line uses equations (6) and (7), the third line is simple algebra, the fourth line follows from the fact that $N_{\text{AT}}\left(x\right)-1=N_{\text{AT}}\left(y\right)$ if *x* can be converted to *y* by an A→C mutation, the fifth line follows from equation (5), and the final line again substitutes the definitions of equations (10) and (4).

Taken together, equations (14)–(17) establish that equation (13) holds for all possible independent types of mutations.

Finally, to show that the overall evolutionary model in equation (1) is reversible and has $p_{r,x}$ defined by equation (9) as its equilibrium frequency, it is necessary to show that $p_{r,x}\times P_{r,xy}=p_{r,y}\times P_{r,yx}$. This follows trivially from equations (12) and (13):

(18) $$\begin{array}{c} \;p_{r,x}\times P_{r,xy}=\frac{{\left(\pi_{r,A\left(x\right)}\right)}^{\beta }\times q_{x}}{\sum_{z}{\left(\pi_{r,A\left(z\right)}\right)}^{\beta }\times q_{z}}\times \left(Q_{xy}\times F_{r,xy}\right)\\ \;=\frac{\left({\left(\pi_{r,A\left(x\right)}\right)}^{\beta }\times F_{r,xy}\right)\times \left(q_{x}\times Q_{xy}\right)}{\sum_{z}{\left(\pi_{r,A\left(z\right)}\right)}^{\beta }\times q_{z}}\\ \;=\frac{\left({\left(\pi_{r,A\left(y\right)}\right)}^{\beta }\times F_{r,yx}\right)\times \left(q_{y}\times Q_{yx}\right)}{\sum_{z}{\left(\pi_{r,A\left(z\right)}\right)}^{\beta }\times q_{z}}\\ \;=\frac{{\left(\pi_{r,A\left(y\right)}\right)}^{\beta }\times q_{y}}{\sum_{z}{\left(\pi_{r,A\left(z\right)}\right)}^{\beta }\times q_{z}}\times \left(Q_{yx}\times F_{r,yx}\right)\\ \;=p_{r,y}\times P_{r,yx}. \end{array}$$

The fact that $P_{r,xy}$ defines a reversible Markov process with stationary state $p_{r,x}$ means that it is possible to define a symmetric matrix $S_{r}$ such that

(19) $$S_{r}diag\left(\ldots ,p_{r,x},\ldots \right)=P_{r},$$

where $diag\left(\ldots ,p_{r,x},\ldots \right)$ is the diagonal matrix with $p_{r,x}$ along its diagonal. Noting $S_{r}=P_{r}diag\left(\ldots ,\frac{1}{p_{r,x}},\ldots \right)$, we have
(20)
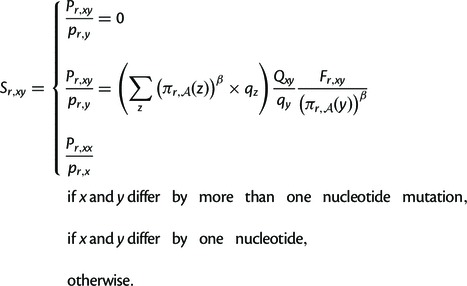
 This matrix is symmetric because $S_{r,xy}=S_{r,yx}$ as can be verified from the fact that $\frac{Q_{xy}}{q_{y}}=\frac{Q_{yx}}{q_{x}}$ and $\frac{F_{r,xy}}{{\left(\pi_{r,A\left(y\right)}\right)}^{\beta }}=\frac{F_{r,yx}}{{\left(\pi_{r,A\left(x\right)}\right)}^{\beta }}$ as is guaranteed by equations (12) and (13).

## Supplementary Material

Supplementary files S1–S4 are available at *Molecular Biology and Evolution* online (http://www.mbe.oxfordjournals.org/).

Supplementary Data
