# Supplementary material for: An Experimentally Informed Evolutionary Model Improves Phylogenetic Fit to Divergent Lactamase Homologs
Source: Mol Biol Evol. 2014 Jul 24;31(10):2753–69. doi: 10.1093/molbev/msu220 (PMC4166927; doi:10.1093/molbev/msu220)
Supplement: Supplementary Data [file supp_31_10_2753__index.html]

An experimentally informed evolutionary model improves phylogenetic fit to divergent lactamase homologs — An Experimentally Informed Evolutionary Model Improves Phylogenetic Fit to Divergent Lactamase Homologs — An Experimentally Informed Evolutionary Model Improves Phylogenetic Fit to Divergent Lactamase Homologs — Supplementary Data 

# An Experimentally Informed Evolutionary Model Improves Phylogenetic Fit to Divergent Lactamase Homologs

## Supplementary Data

files

**Files in this Data Supplement:**

- Supplementary Data - txt file
- Supplementary Data - txt file
- Supplementary Data - txt file
- Supplementary Data - txt file
